# Supplementary material for: Reduction in maternal mortality ratio varies by district in Sidama National Regional State, southern Ethiopia: Estimates by cross-sectional studies using the sisterhood method and a household survey of pregnancy and birth outcomes
Source: PLoS One. 2023 Oct 12;18(10):e0276144. doi: 10.1371/journal.pone.0276144 (PMC10569508; doi:10.1371/journal.pone.0276144)
Supplement: S1 Checklist — (DOCX) [file pone.0276144.s001.docx]

**S1 Checklist. STROBE Statement**

STROBE Statement—checklist of items that should be included in reports of observational studies

|  | Item No. | Recommendation | Page  No. | Relevant text from manuscript |
| --- | --- | --- | --- | --- |
| **Title and abstract** | 1 | (*a*) Indicate the study’s design with a commonly used term in the title or the abstract | 1 | Reduction in maternal mortality ratio varies by district in Sidama Regional State, southern Ethiopia: Estimates by cross-sectional studies using the sisterhood method and a household survey of pregnancy and birth outcomes |
|  |  | (*b*) Provide in the abstract an informative and balanced summary of what was done and what was found | 2-3 | **Background:** Maternal mortality studies conducted at national level do not provide estimates useful for monitoring maternal mortality at lower administrative level.  **Objective**: The aim of this study was to estimate life time risk (LTR) of maternal death and maternal mortality ratio (MMR) and assess reduction in MMR.  **Design and setting:** This is population based cross-sectional study conducted in Sidama Regional State, southern Ethiopia from July 2019 to May 2020.  **Participants**: men and women aged 15-49 years  **Analysis:** By creating a retrospective cohort of women in reproductive age, we calculated LTR of maternal mortality and approximated the MMR using the total fertility for the rural Ethiopian population. Variations in maternal mortality was assessed based on characterstics of the respondents. Reduction in MMR was examined using the estimates of the sisterhood method and 5-year recall of pregnancy and birth outcome household survey.  **Results:** We analysed 17374 (99.6%) respondents; 8884 (51.1%) men and 8490 (48.9%) women. The 17,374 respondents reported 64,387 maternal sisters. 2,402 (3.7%) sisters had died; 776 (32.3%) were pregnancy related deaths. The LTR of maternal death was 3.2% and MMR was 623 (95% CI: 573-658) per 100000 live births (LB). The remote district (Aroresa) had a MMR of 1210 (95% CI: 1027-1318) per 100,000 LB. The estimate from male and female respondents was not different. Significant reduction in MMR was observed in districts located near the regional centre. However, no reduction was observed in districts located distant to the regional centre.  **Conclusions:** The high MMR with district level variations and lack of mortality reduction in districts located distant to the centre highlights the need for instituting interventions tailored to the local context to save mothers and accelerate reduction in MMR. |
| Introduction | | | |  |
| Background/rationale | 2 | Explain the scientific background and rationale for the investigation being reported | 3-4 | The data used for monitoring maternal mortality in the country largely comes from Demographic and Health Surveys and United Nations agency reports conducted periodically at national level. However, national level studies do not provide data on magnitude and variations of maternal deaths at sub-national and district level useful for program planning and monitoring maternal mortality at lower administrative level |
| Objectives | 3 | State specific objectives, including any prespecified hypotheses | 4-5 | Our specific objectives in this study using the indirect sisterhood method were 1) to estimate life time risk (LTR) of maternal death and corresponding MMR; 2) to assess variations in maternal mortality estimates based on characterstics of the respondents; 3) to assess reduction in MMR in Sidama Regional State over the past years by using MMR of the sisterhood method (main focus of this paper) with results from the 5-year recall of pregnancy and birth outcome household survey. |
| Methods | | | |  |
| Study design | 4 | Present key elements of study design early in the paper | 5 | We carried out population based cross sectional study employing the sisterhood method (main focus of this paper) as part of larger maternal mortality household survey that used a retrospective 5-year recall of pregnancy and birth outcomes |
| Setting | 5 | Describe the setting, locations, and relevant dates, including periods of recruitment, exposure, follow-up, and data collection | 5 | The study was conducted in Sidama Regional State, Southern Ethiopia. in randomly selected 6 (20%) districts (Aleta Chuko, Aleta Wondo, Aroresa, Daela, Hawassa Zuriya and Wondogenet) of the region. The study was conducted from July 2019 to May 2020. Sidama Regional State is one of 11 regional states in the country, and its capital Hawassa is located 273 km south of Addis Ababa. The population of the region projected for 2020 was 4.3 million people. Administratively, the region is divided into 30 rural districts (woredas), 6 town administrations and 536 rural kebeles (the smallest administrative structure with an average population of 5000 people). Under the kebele, there are local structure known as limatbudin (local administrative unit consisting of 40-50 neighbouring households in average). |
| Participants | 6 | (*a*) *Cohort study*—Give the eligibility criteria, and the sources and methods of selection of participants. Describe methods of follow-up  *Case-control study*—Give the eligibility criteria, and the sources and methods of case ascertainment and control selection. Give the rationale for the choice of cases and controls  *Cross-sectional study*—Give the eligibility criteria, and the sources and methods of selection of participants | 6-7 | Men and women aged 15-49 years in Sidama Regional State were source population of the study, and men and women of same age and residing in sampled households were the study population.  In the sampled households, the data collectors interviewed the husband, wife, children and any family member aged 15-49 years. When two or more eligible participants born to the same mother were found in the same household, one of them was chosen by a lottery method to avoid multiple counting. |
|  |  | (*b*) *Cohort study*—For matched studies, give matching criteria and number of exposed and unexposed  *Case-control study*—For matched studies, give matching criteria and the number of controls per case |  |  |
| Variables | 7 | Clearly define all outcomes, exposures, predictors, potential confounders, and effect modifiers. Give diagnostic criteria, if applicable | 7 | Life time risk of maternal deaths: the probability that a 15-year-old female will die eventually from a maternal cause and MMR; maternal deaths per 100,000 LB were the outcome measures of the study. We also collected characterstics of the respondents, like sex, age and educational level to assess their association with the outcome measures of interest. |
| Data sources/ measurement | 8* | For each variable of interest, give sources of data and details of methods of assessment (measurement). Describe comparability of assessment methods if there is more than one group | 7-8 | The data collectors conducted an interview with men and women 15-49 years using interviewer administered questionnaire. Participants were asked the following four standard questions used in indirect sisterhood method. 1) How many sisters (born to the same mother) have you ever had who reached 15 years? 2) How many of these sisters who ever-reached 15 years are alive now? 3) How many of these sisters who ever-reached 15 years are dead? 4) How many of these dead sisters died while they were pregnant, or during childbirth, or during the six weeks after the end of pregnancy?  The age of the participants was obtained directly asking the participants about their age and verified by the interviewers using key local and national events. Completed grade or education level was used to label the highest educational level attained and those who cannot read and write were labelled as having “no formal education” |
| Bias | 9 | Describe any efforts to address potential sources of bias | 6-7 | When two or more eligible participants born to the same mother were found in the same household, one of them was chosen by a lottery method to avoid multiple counting. |
| Study size | 10 | Explain how the study size was arrived at | 9 | Hanley and colleagues suggested that, in settings where the MMR within the range of 500 per 100,000 LB, the death of 385 sisters with (± 10% margin of error) will be reported from interviews of 13000 adult respondents aged 15-49. According to the 2016 EDHS report, the MMR of Ethiopia was 412/100,000 LB. Adding 10% non-responses, we decided to interview 14,300 respondents. The number of households planned for the larger maternal mortality household survey was 8880. We assumed that, surveying the 8880 household would be sufficient to get the desired sample of adult respondents for the sisterhood study. |

Continued on next page

| Quantitative variables | 11 | Explain how quantitative variables were handled in the analyses. If applicable, describe which groupings were chosen and why | 9 | Before LTR and MMR computation, two adjustments were made by grouping the participants’ age in five age group to estimate the number of sisters would be reported by the younger age group and to get the number of sisters exposed to full time risk exposure at each age group |
| --- | --- | --- | --- | --- |
| Statistical methods | 12 | (*a*) Describe all statistical methods, including those used to control for confounding | 10 | Descriptive statistics with means and percentages were computed to describe participants’ characteristics. The LTR for maternal death was obtained by dividing the total number of maternal deaths reported by the estimated total number of sisters exposed (LTR= (Total number of maternal deaths)/ (Total sister units of risk exposure). We used total fertility rate (TFR) of rural population of Ethiopia 5.2 to estimate MMR from the LTR. (MMR = (1-[(1- LTR) 1/TFR] x 100,000), using formulas specified by Hanley et al.  The corresponding time period to which our estimate refers was computed using the following formula: T = Σ(T(i)*B(i))/ΣB(i), where T = the point time location of the global estimate, T(i) = the time location of the estimate for each age group and B(i) = the exposing units of each age group. We carried out stratified analysis based on respondents’ sex, age, and location to see the association with the outcome measure. Finally, we assessed differences and reduction in MMR in Sidama Regional State and in the districts included in this study using the two maternal mortality estimations methods: the sisterhood method and the 5-year recall of pregnancy and birth outcome household survey |
|  |  | (*b*) Describe any methods used to examine subgroups and interactions | 10 | We carried out stratified analysis based on respondents’ sex, age, and location to see the association with the outcome measure. |
|  |  | (*c*) Explain how missing data were addressed | 11 | Seventy (0.4%) respondents had incomplete information and were excluded from the analysis. There was no reported death of sisters from the 70 respondents with incomplete information. |
|  |  | (*d*) *Cohort study*—If applicable, explain how loss to follow-up was addressed  *Case-control study*—If applicable, explain how matching of cases and controls was addressed  *Cross-sectional study*—If applicable, describe analytical methods taking account of sampling strategy |  |  |
|  |  | (*e*) Describe any sensitivity analyses |  |  |
| Results | | | | |
| Participants | 13* | (a) Report numbers of individuals at each stage of study—eg numbers potentially eligible, examined for eligibility, confirmed eligible, included in the study, completing follow-up, and analysed | 11 | We conducted an interview with 17,444 men and women 15-49 years of age residing in 8880 households. Seventy (0.4%) respondents had incomplete information and were excluded from the analysis. There was no reported death of sisters from the 70 respondents with incomplete information. The final analysis included 17,374 (99.6%) respondents: 8,884 (51.1%) men and 8,490 (48.9%) women. |
|  |  | (b) Give reasons for non-participation at each stage |  | None |
|  |  | (c) Consider use of a flow diagram |  | Not Applicable |
| Descriptive data | 14* | (a) Give characteristics of study participants (eg demographic, clinical, social) and information on exposures and potential confounders | 11-12 | The mean age of the respondents was 29.3 years (SD=6.8), 20.5% had no formal education and 15.2% had attended high school or higher education.  Table 1. Characterstics of respondents of maternal mortality survey using the sisterhood method, in Sidama Regional State, southern Ethiopia, 2020 |
|  |  | (b) Indicate number of participants with missing data for each variable of interest |  | None |
|  |  | (c) *Cohort study*—Summarise follow-up time (eg, average and total amount) |  |  |
| Outcome data | 15* | *Cohort study*—Report numbers of outcome events or summary measures over time |  |  |
|  |  | *Case-control study—*Report numbers in each exposure category, or summary measures of exposure |  |  |
|  |  | *Cross-sectional study—*Report numbers of outcome events or summary measures | 12-13 | The total LTR of maternal death was 3.2% i.e. 1 in 31 women 15-49 years old dies due to maternal cause. Using total fertility rate of 5.2, the estimated MMR for the study area was 623 (95% CI: 573-658) per 100,000 LB. |
| Main results | 16 | (*a*) Give unadjusted estimates and, if applicable, confounder-adjusted estimates and their precision (eg, 95% confidence interval). Make clear which confounders were adjusted for and why they were included |  |  |
|  |  | (*b*) Report category boundaries when continuous variables were categorized |  |  |
|  |  | (*c*) If relevant, consider translating estimates of relative risk into absolute risk for a meaningful time period |  |  |

| Other analyses | 17 | Report other analyses done—eg analyses of subgroups and interactions, and sensitivity analyses | 13, 14, 15, 16 | The MMR among participants in the 15-29 age group was 545 (95% CI: 475-601) per 100, 000 LB, reflecting 7 years before data collection. For respondents in the 30-39 age group, the MMR was 703 (624-760) per 100, 000 LB, reflecting a period 11 years before the study. For respondents 40 years or more, the MMR was 564 (455-660) per 100, 000 LB, reflecting a period 15 years before data collection.  Table 3B shows the life time risk of maternal death and MMR stratified by male and female respondents. There was no statistically significant difference among male and female respondents.  Table 3C shows stratified analysis of maternal mortality estimates by the districts of respondents. The MMR in Aroresa district was significantly higher than all other study districts in the region; MMR: 1210 (95% CI: 1027-1318) per 100,000 LB.  The sisterhood method refers to around 10 years before the study and showed the overall MMR of 623/100,000 LB in Sidama Regional State. The survey with 5yr recall found 419/100,000 LB with slightly overlapping confidence intervals. Some districts had lower MMR in the survey of 5yr pregnancy recall than the sisterhood estimates reflecting 10 years back, whereas others like Aroresa had similar estimates in both. |
| --- | --- | --- | --- | --- |
| Discussion | | | | |
| Key results | 18 | Summarise key results with reference to study objectives | 17 | By incorporating the sisterhood method in a household survey of pregnancy and birth outcomes, we found a lifetime risk of maternal death of 3.2 %, with a corresponding MMR of 623 per 100,000 LB; the time reference was 2010. The remote district (Aroresa) had significantly higher MMR. Sub analysis of MMR based on the respondents’ age showed that participants 15-29 years of age had a MMR; 545 per 100, 000 LB, reflecting 7 years before data collection. Stratified analysis for male and female respondents provided similar maternal mortality estimate.  MMR estimated by a household survey that used a 5-year recall of pregnancy found an estimated MMR of 419 per 100,000 LB and the sisterhood method referring to 10 years before the study found 623 per 100,000 LB, slightly overlapping confidence intervals. Districts located distant from the centre with poor infrastructure and inadequate skilled health personnel did had similar MMR by both methods and seem to have persistently high MMR. Whereas, districts located near to the centre with good infrastructure and adequate skilled health personnel have lower estimated MMR by the 5-year recall than by the sisterhood method. |
| Limitations | 19 | Discuss limitations of the study, taking into account sources of potential bias or imprecision. Discuss both direction and magnitude of any potential bias | 18-19 | This study had some limitations similar to other studies employed the sisterhood method. The maternal mortality estimates we reported refer a period around 10 years before data collection; thus we were not able to show recent estimates using this method.  There might be over-reporting of deaths. Over reporting of deaths may arise due to inclusion of deaths that occurred beyond six weeks of end of pregnancy or cases which were not related to pregnancy. Multiple counting could also be the reason for over-estimation of maternal deaths using the sisterhood method.  To minimize over-reporting, we used data collectors who were familiar with language and culture of the study population. As a result of familiarity with local context, the data collectors supported participants in identifying pregnancy state of deceased sisters and time of their deaths. In addition, in households where more than one eligible participants born to the same mother were found, we chose one of them by a lottery method which minimized multiple counting. Despite our effort, there might be over-reporting in our study.  Underreporting could also be another limitation for this study. Deaths occurring at early stage of pregnancy due to abortion or ectopic pregnancy and deaths of women not in marital union might not be reported.  We did not have information on place of living for the sisters in the cohort. We used the respondents’ residences as proxy for the sisters’ location. Some sisters might have moved to other districts. Our study also lack information on age at death of respondents’ sisters but used respondents’ age to observe recent deaths and show patterns of deaths across age groups. Age of the respondents may not closely reflect the age of their sisters. |
| Interpretation | 20 | Give a cautious overall interpretation of results considering objectives, limitations, multiplicity of analyses, results from similar studies, and other relevant evidence | 19, 20, 21 | We found high MMR in the study area with time reference in 2010. Our result is significantly higher than the findings of national DHS [6], which reported a MMR 412 per 100,000 LB (time reference, 2009-2016)…..  The estimate from our study was also higher than a study from Kersa Health and Demographic Surveillance Site (HDSS) [16], which estimated a MMR of 396 per 100,000 LB for the year 2010. The variation could be explained by the differences of methods the two studies used for MMR estimation, access to maternal health care and differences in documentation of maternal deaths.  In our study, we found similar maternal mortality estimates both from men and women respondents….  This study found high maternal mortality estimates among the respondents in the 30-39 age group; MMR: 703 per 100,000 LB compared to participants in 15-29 years of age. Our finding is different from findings reported from Nigeria where they found the highest MMR among the respondents in the 15-29 age group [10]….  Our study has also shown significant variations in MMR by districts of the respondents. This is in agreement with the findings of study conducted in northern Nigeria and Mali [9, 10]…..  The high MMR observed in Aroresa districts (the remote district) could be explained by poor road facilities and difficult topography that might hamper access to health services. Aroresa district is situated 181 km away from the regional centre [18]….  In our study of Hawassa Zuriya district (the central district), the sisterhood study reflecting 10 years back gave a MMR of 707 and 5y recall gave 114 per 100.000 LB. |
| Generalisability | 21 | Discuss the generalisability (external validity) of the study results | 22 | We considered the size of the sample and sampling techniques while conducting the studies using the two maternal mortality estimation methods in order to precisely estimate the MMR in the region. Hence, representative and large number of participants were included in both 5-year recall of pregnancy and birth outcome household survey and the sisterhood study. In 5-year recall of pregnancy and birth outcome household survey, we registered 10602 LB and 48 maternal deaths in 8880 households visited. Our aim was to find 66 maternal deaths with MMR; 412 (95% CI: 324-524) per 100,000 LB. The MMR after the study was 419 (95% CI: 260-577) which is within the 95% CI we anticipated initially [8]. For the sisterhood study, we had an interview with 17444 siblings. This sample size was above the recommended 13,000 siblings for the indirect sisterhood study in settings with similar magnitude of maternal mortality [13].The samples for both studies were selected using probability sampling and multistage cluster sampling technique was employed to select the study participants. |
| Other information | |  | | |
| Funding | 22 | Give the source of funding and the role of the funders for the present study and, if applicable, for the original study on which the present article is based |  | Detailed in the additional submission required information. |

*Give information separately for cases and controls in case-control studies and, if applicable, for exposed and unexposed groups in cohort and cross-sectional studies.

**Note:** An Explanation and Elaboration article discusses each checklist item and gives methodological background and published examples of transparent reporting. The STROBE checklist is best used in conjunction with this article (freely available on the Web sites of PLoS Medicine at http://www.plosmedicine.org/, Annals of Internal Medicine at http://www.annals.org/, and Epidemiology at http://www.epidem.com/). Information on the STROBE Initiative is available at www.strobe-statement.org.
