# Supplementary material for: Reduction in maternal mortality ratio varies by district in Sidama National Regional State, southern Ethiopia: Estimates by cross-sectional studies using the sisterhood method and a household survey of pregnancy and birth outcomes
Source: PLoS One. 2023 Oct 12;18(10):e0276144. doi: 10.1371/journal.pone.0276144 (PMC10569508; doi:10.1371/journal.pone.0276144)
Supplement: S2 Checklist — (DOCX) [file pone.0276144.s002.docx]

Inclusivity in global research

PLOS’ policy on inclusivity in global research aims to improve transparency in the reporting of research performed outside of researchers’ own country or community and ensures that PLOS publications reporting global research adhere to high standards for research ethics and authorship. Authors of relevant research articles may be asked to complete the questionnaire below, which outlines ethical, cultural, and scientific considerations specific to inclusivity in global research. This questionnaire may be requested when researchers have travelled to a different country to conduct research, if research uses samples collected in another country, research with Indigenous populations or their lands, or if research is on cultural artefacts. Researchers travelling to another country solely to use laboratory equipment will not normally be required to complete the questionnaire. However, the questionnaire can be requested at the journal’s discretion for any submission – if you have been requested to complete this questionnaire by the PLOS journal you submitted to, please do so.

Please complete the questionnaire below and include this as a Supporting Information file with your manuscript. Note that if your paper is accepted for publication, this checklist will be published with your article in the supporting information files. Please ensure that you reference the checklist in the main body of your manuscript. We suggest adding a subsection ‘Inclusivity in global research’ to your Methods section and adding the following sentence: “Additional information regarding the ethical, cultural, and scientific considerations specific to inclusivity in global research is included in the Supporting Information (SX Checklist)”

The questions have been designed to be applicable to a wide range of study types, and there are subsections for both human subjects research and non-human subjects research. If any of the questions are not relevant to your research please mark them as “N/A” as appropriate.

**Ethical considerations, permits and authorship**

*This section is applicable to all research types.*

Provide details as to who granted permissions and/or consent for the study to take place in the Methods section of your manuscript. This should include the names of **all** ethics boards, governmental organizations, community leaders or other bodies that provided approval for the study. If individuals provided approval refer to these people by their role or title but do not list their name(s).

Reported on page number: 11

If there were any deviations from the study protocol after approval was obtained please provide details of these changes in the Methods section of your manuscript.

Reported on page number: N/A

Did this study involve local collaborators that are residents of the country where the research was conducted or members of the community studied? If you do not have any authors from said communities, please provide an explanation for this below.
Everyone listed as an author should meet PLOS’ criteria for authorship and all individuals who meet these criteria should be included in the author byline, rather than the acknowledgements. For further information please see the journal’s Authorship Policy.

I, the principal investigator (AZK), and one of the co-authors of the study (AGT), are residents of the Sidama National Regional State where the study has been conducted. Moreover, AZK, BL, and AGT are affiliated with Hawassa University in Ethiopia, located in the Sidama Region.

**Human subjects research (e.g. health research, medical research, cross-cultural psychology)**

Did you obtain written informed consent from a representative of the local community or region before the research took place? How did you establish who speaks for the community? Details of written informed consent obtained from study participants should be reported separately in the Methods section of your manuscript.

We obtained support letters from the Sidama National Regional State Health Bureau (formerly known as Sidama Zone Health Department), district health offices, and kebele (village) administrations. The Kebele administration was responsible for speaking to sub-village community representatives. We have reported the details of the consent we obtained from the participants in the method section.

How did members of the local community provide input on the aims of the research investigation, its methodology, and its anticipated outcome(s)?

We obtained the updated list of the districts and kebeles from the Regional Health Bureau and the district health offices, respectively. Finally, we got the list of limatbudins (sub-villages) from the Kebele administration. All the local community structures we mentioned above supported our research methodology (sampling strategy) and the anticipated results.

When engaging with the local community, how did you ensure that the informed consent documents and other materials could be understood by local stakeholders?

We informed the participants that their participation is voluntary, and they are able to withdraw or leave the study at any time. We told the participant that there were no negative consequences or repercussions for their refusal to participate. We also provided the participants with the following information: what the study is about, the risks and benefits of taking part, and how long the study will take. We informed the participants that their personal identifiers would not be collected, and the information collected from them would not be accessible or shared with anybody except the designated research team. We used a consent form written in a local language. Finally, for those who are able to read and write, we provided copies of the consent form and asked if they had any questions. For those who were not able to read and write, we explained the consent form verbally. After they agreed to participate, we made them sign the consent form (written or thumb) with a date on it.

Will the findings of the research be made available in an understandable format to stakeholders in the community where the study was conducted (e.g. via a presentation, summary report, copies of publications, etc.)? Please provide details of how this will be achieved.

The summary of the findings of the study will be presented at the regional health bureau and ministry of health annual meetings. The copies of the publication will be shared with the regional health bureau, the maternal and child health directorate at the regional health bureau, and the respective district health offices.

**Non-human subjects research using specimens/ animals collected as part of the study, or those housed in archival collections. Examples include archaeology, paleontology, botany and zoology.**

Did the permission you obtained from a local authority to perform the study include an agreement on access to outputs and benefit sharing? This may include procedures to enable fair distribution of the benefits and resources arising from the research performed. Please include any details of Prior Informed Consent and Benefit Sharing Agreements obtained. These may be required by field-specific regulations, for example the Convention on Biological Diversity (CBD) and the associated Nagoya Protocol.

N/A

If the material used in your study was imported, please A) provide the year it was imported and B) indicate whether permits were obtained to import/export the materials used, C) provide details of any permits obtained. If this information is not available, please indicate this.

N/A

If you used archival specimens, please state how the material used in your study was acquired by the institute it is held in and provide details of any permits obtained for the original excavations/ sample collection. If this information is not available, please indicate this.

N/A

How was the potential cultural significance of the materials collected in your study to local communities considered in your research design? Were Indigenous peoples and/or local researchers and institutions involved with archaeological excavations / collection of specimens? If so, please provide a description of their involvement.

N/A

If your manuscript includes photographs of human remains please indicate whether authors obtained permission from descendants or affiliated cultural communities to do so.

N/A
